# Targeting metabotropic glutamate receptors for novel treatments of schizophrenia

**DOI:** 10.1186/s13041-017-0293-z

**Published:** 2017-04-26

**Authors:** James Maksymetz, Sean P. Moran, P. Jeffrey Conn

**Affiliations:** 1 Department of Pharmacology, Vanderbilt University, Nashville, TN 37232 USA; 2 Vanderbilt Center for Neuroscience Drug Discovery, Nashville, TN 37232 USA; 3 Vanderbilt Brain Institute, Vanderbilt University, Nashville, TN 37232 USA

**Keywords:** mGlu receptor, Schizophrenia, Allosteric modulator, Signal bias, Heterodimer, PAM, NAM, Glutamate

## Abstract

Support for the N-methyl-d-aspartate receptor (NMDAR) hypofunction hypothesis of schizophrenia has led to increasing focus on restoring proper glutamatergic signaling as an approach for treatment of this devastating disease. The ability of metabotropic glutamate (mGlu) receptors to modulate glutamatergic neurotransmission has thus attracted considerable attention for the development of novel antipsychotics. Consisting of eight subtypes classified into three groups based on sequence homology, signal transduction, and pharmacology, the mGlu receptors provide a wide range of targets to modulate NMDAR function as well as glutamate release. Recently, allosteric modulators of mGlu receptors have been developed that allow unprecedented selectivity among subtypes, not just groups, facilitating the investigation of the effects of subtype-specific modulation. In preclinical animal models, positive allosteric modulators (PAMs) of the group I mGlu receptor mGlu_5_ have efficacy across all three symptom domains of schizophrenia (positive, negative, and cognitive). The discovery and development of mGlu_5_ PAMs that display unique signal bias suggests that efficacy can be retained while avoiding the neurotoxic effects of earlier compounds. Interestingly, mGlu_1_ negative allosteric modulators (NAMs) appear efficacious in positive symptom models of the disease but are still in early preclinical development. While selective group II mGlu receptor (mGlu_2/3_) agonists have reached clinical trials but were unsuccessful, specific mGlu_2_ or mGlu_3_ receptor targeting still hold great promise. Genetic studies implicated mGlu_2_ in the antipsychotic effects of group II agonists and mGlu_2_ PAMs have since entered into clinical trials. Additionally, mGlu_3_ appears to play an important role in cognition, may confer neuroprotective effects, and thus is a promising target to alleviate cognitive deficits in schizophrenia. Although group III mGlu receptors (mGlu_4/6/7/8_) have attracted less attention, mGlu_4_ agonists and PAMs appear to have efficacy across all three symptoms domains in preclinical models. The recent discovery of heterodimers comprising mGlu_2_ and mGlu_4_ may explain the efficacy of mGlu_4_ selective compounds but this remains to be determined. Taken together, compounds targeting mGlu receptors, specifically subtype-selective allosteric modulators, provide a compelling alternative approach to fill the unmet clinical needs for patients with schizophrenia.

## Introduction

Schizophrenia is a devastating psychiatric disorder that afflicts approximately 1% of the worldwide population, affects women and men equally, and spans all socioeconomic groups [1]. The disease is characterized by three major symptom domains: positive, negative, and cognitive symptoms [2]. Current antipsychotics are effective at treating the positive symptoms such as auditory and visual hallucinations, delusions, and disorganized thoughts; however, they do not address the negative nor the cognitive symptoms. Negative symptoms (e.g., flattened affect, social withdrawal) and cognitive symptoms (e.g., deficits in working memory, and cognitive flexibility) are believed to be the best predictors of long-term outcome and are estimated to cost the U.S. healthcare system over $60 billion per year [3–5]. Additionally, most patients discontinue current treatments due to adverse effects including extrapyramidal side effects (EPS) (i.e., dystonia, akathisia, parkinsonism, bradykinesia, tremor, and tardive dyskinesia) induced by first-generation typical antipsychotics and metabolic side effects (i.e., weight gain, type II diabetes, and hyperlipidosis) induced by second generation atypical antipsychotics [6–8].

While most current antipsychotics act by antagonizing the hyperdopaminergic and hyperserotonergic states underlying the positive symptoms, there is a growing body of evidence that supports glutamate dysfunction as a contributing factor for the disease [9, 10]. For example, administration of the N-methyl-D-aspartate receptor (NMDAR) antagonist phencyclidine (PCP) [11] induces a schizophrenia-like state that presents clinically with all three symptom clusters in healthy individuals [12, 13]. Similar clinical results have been found with administration of other NMDAR antagonists such as ketamine [14]. NMDAR antagonists also exacerbate or precipitate controlled symptoms when administered to schizophrenia patients [15]. This along with extensive preclinical evidence suggests that NMDAR hypofunction is important in the pathophysiology underlying schizophrenia [10].

Based on the NMDAR hypofunction hypothesis of schizophrenia, pharmacological agents that enhance NMDAR function are not only valuable tools in preclinical animal models but could also provide therapeutic benefits to patients with schizophrenia. Unfortunately, direct activation of NMDARs using traditional orthosteric agonists induces adverse effects such as excitotoxicity and seizures [16–20]. Furthermore, treatments with NMDAR obligate co-agonists such as glycine or serine failed to have consistent efficacy across multiple clinical trials [21]. More recently, selective NMDAR positive allosteric modulators (PAMs) that enhance receptor function in the presence of the endogenous agonists but are devoid of intrinsic activity have been reported [20]. It is possible that NMDAR PAMs could avoid the adverse effects associated with direct activation of NMDARs. The recent development of NMDAR PAMs such as GNE-6901 and GNE-8324 provide proof-of-principle for the development of allosteric modulators of NMDARs, however their poor pharmacokinetic properties and low central nervous system exposures hinder their uses for in vivo studies [20]. Therefore, it will be important to develop more optimized compounds to fully assess the ability of NMDAR PAMs to reverse schizophrenia-like symptoms in animal models without the adverse effects profile of NMDAR agonists.

In addition to NMDARs and other ionotropic glutamate receptors (α-amino-3-hydroxy-5-methyl-4-isoxazolepropionic acid (AMPA) and kainate receptors) that mediate fast excitatory neurotransmission, glutamate also binds to and signals through a family of G-protein coupled metabotropic glutamate (mGlu) receptors [22]. There are eight subtypes of mGlu receptors, mGlu_1–8_, classified into three groups (group I, mGlu_1,5_; group II, mGlu_2,3_; group III, mGlu_4,6,7,8_). mGlu receptors are class C GPCRs which function primarily as dimers and modulate glutamatergic, GABAergic, and neuromodulatory neurotransmission throughout the central nervous system (CNS) [22]. All three groups of mGlu receptors have been pursued as putative targets for novel antipsychotics due to their ability to directly alter NMDAR function or other aspects of glutamatergic signaling.

The highly conserved orthosteric glutamate binding site among mGlu receptor subtypes has generally precluded the design of subtype-specific receptor agonists or antagonists. This problem has been addressed by designing small molecule modulators that bind to distinct and subtype-unique allosteric sites within the 7 transmembrane domains of mGlu receptors allowing unprecedented selectivity against other glutamate receptors [23]. Positive allosteric modulators (PAMs) generally do not activate the receptor directly but instead potentiate responses to endogenous glutamate. Conversely, negative allosteric modulators (NAMs) act as non-competitive antagonists and may also have inverse agonist activity, reducing constitutive activity of the receptor in the absence of glutamate [24, 25].

## Group I mGlu receptors (mGlu_1_ & mGlu_5_)

**Fig. 1:**
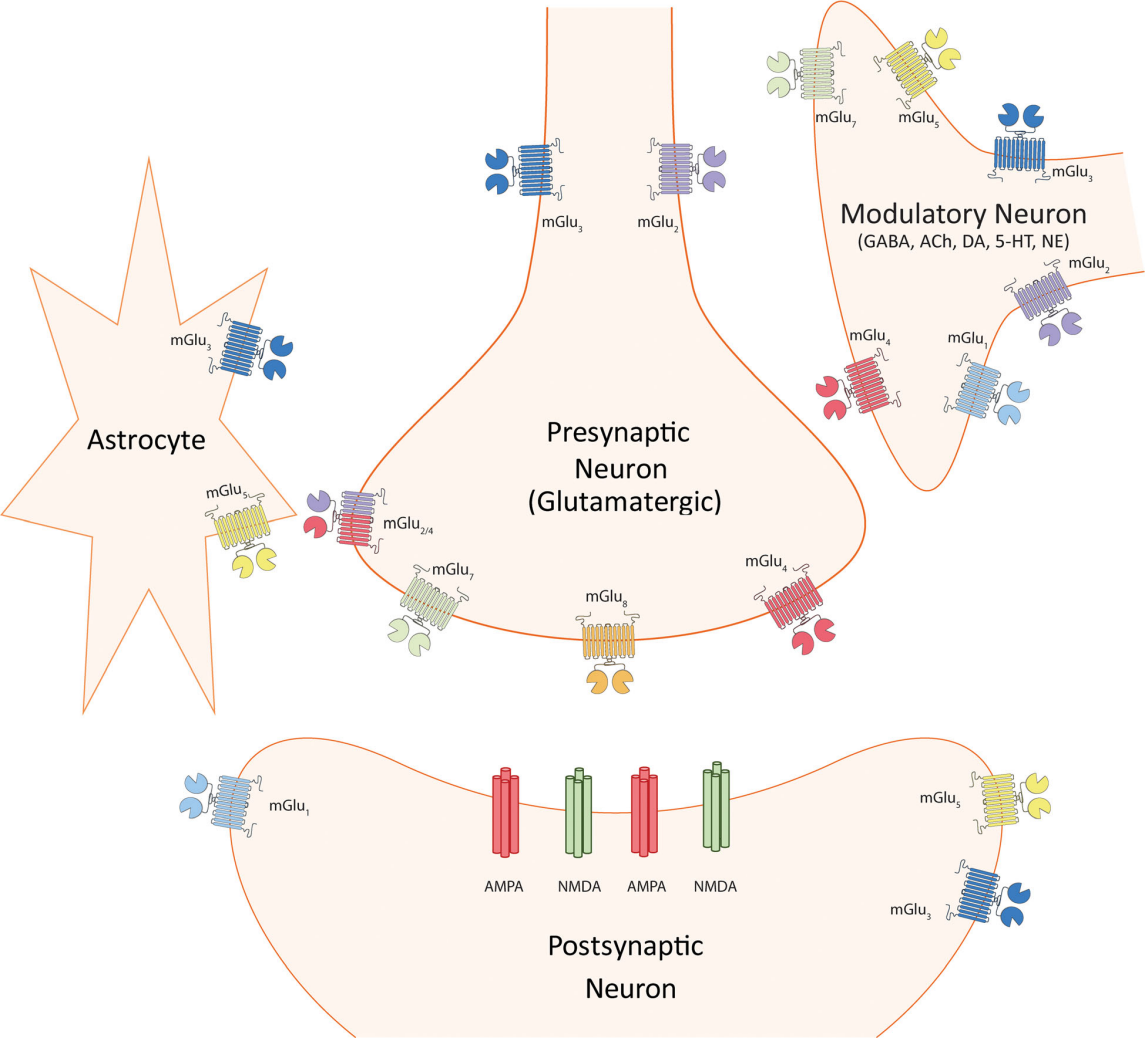
Localization of metabotropic glutamate receptor subtypes. Schematic representation of the predominant locations of mGlu receptors at the synapse. mGlu_1_ (*light blue*) is found on postsynaptic glutamatergic neurons as well as on GABAergic neurons. mGlu_5_ (*yellow*) can be located on the same neurons as mGlu_1_ as well as on glia. mGlu_2_ (*purple*) is found primarily presynaptically as both a homodimer as well as a heterodimer with mGlu_4_ (*red*). mGlu_3_ (*dark blue*) is found on both presynaptic and postsynaptic glutamatergic, GABAergic, and neuromodulatory neurons as well on glia. mGlu_4_ is localized to both modulatory neurons as well as on presynaptic glutamatergic neurons as either a homodimer or heterodimer. mGlu_7_ (*green*) is localized to presynaptically neurons as well as GABAergic neurons. Lastly, mGlu_8_ (*orange*) is primarily localized presynaptically and mGlu_6_ is not shown since it is restricted to the retina

Group I mGlu receptors include mGlu_1_ [26, 27] and mGlu_5_ [28], and are primarily coupled to the Gα_q_ subunit of the heterotrimeric G-protein. Canonical Gα_q_ signaling activates phospholipase C beta and causes downstream activation of protein kinase C (PKC) via the generation of the second messengers diacylglycerol (DAG) and inositol triphosphate (IP_3_). The group I mGlu receptors have emerged as attractive targets, initially based on their close coupling to the NMDAR via intracellular signaling pathways and scaffolding proteins including Homer, SHANK, and GKAP-PSD95 [29–31] and their ability to potentiate NMDA responses in acute brain slices [32]. mGlu_1_ and mGlu_5_ are predominantly postsynaptic receptors, but they have also been identified on presynaptic terminals of GABAergic and glutamatergic neurons (Fig. 1) [33]. Group I mGlu receptors are found primarily as homodimers via interactions at the large extracellular N-terminal Venus flytrap domain of each monomer [34]. Emerging evidence points to group I mGlu receptors existing in a monomeric form with distinct neurodevelopmental patterns which may impact their pharmacological profiles at specific ages in rodents [35]. While the potential role of dimeric versus monomeric expression may have interesting implications for schizophrenia, further studies are required to investigate this phenomenon.

### mGlu_1_

Two recent independent studies have identified 12 rare deleterious nonsynonymous single nucleotide polymorphisms in the *GRM1* gene encoding for mGlu_1_ in schizophrenia [36, 37]. Further support for mGlu_1_ dysregulation in schizophrenia is evidenced by postmortem findings in which mGlu_1_ mRNA expression is altered compared to controls [38]. Preclinically, *Grm1* knockout mice display deficits in prepulse inhibition (PPI) [39], a behavioral assessment of sensory gating which is the process of filtering unnecessary stimuli from total sensory stimuli and which is impaired in schizophrenia patients [40]. Interestingly, recent studies reveal that *GRM1* mutations associated with schizophrenia reduce mGlu_1_ signaling in cell lines and that selective mGlu_1_ PAMs can partially rescue the reduction in glutamate-mediated calcium signaling in vitro [41]. Therefore, enhancing mGlu_1_ signaling through selective agents has the potential to rescue deficits in schizophrenia patients with deleterious *GRM1* mutations.

In addition to rescuing mGlu_1_ signaling deficits, activators or positive modulators of mGlu_1_ may also act to counteract the hyperdopaminergic signaling in the striatum in schizophrenia patients [42–45]. Multiple studies have demonstrated that the pan-mGlu receptor agonist trans-ACPD is able to attenuate stimulation-induced dopamine release in the dorsal striatum [46], the substantia nigra [47], and the nucleus accumbens [48]. In a follow up study, mGlu_1_ was identified as the subtype responsible for this effect in the dorsal striatum [49]. Therefore, mGlu_1_ activation may have the potential to produce similar antipsychotic effects as D_2_ dopamine receptor antagonist antipsychotics.

Potent first generation mGlu_1_ PAMs were developed in the early 2000s, but poor drug metabolism and pharmacokinetic (DMPK) profiles limited their use in preclinical studies [50, 51]. More recent efforts yielded VU6000799 and VU6000790 as potent, highly selective mGlu_1_ PAMs with improved DMPK properties and brain penetrance, and are therefore better suited for in vivo studies [52–55]. In the future, it will be important to evaluate these compounds in animal models that are relevant to the three symptom domains of schizophrenia.

Important to the potential utility of mGlu_1_ PAMs to treat schizophrenia, it has been reported that the mGlu_1_ NAMs FTIDC and CFMTI are efficacious in animal models predictive of antipsychotic activity (Table 1), including reducing psychostimulant and NMDAR antagonist-induced hyperlocomotion and deficits in PPI as well as reversing deficits in social interaction induced by the NMDAR antagonist MK-801 in rats [56–58]. The contrasting findings of mGlu_1_ PAMs, NAMs, and *GRM1* knockout animals illustrate the potential complexity of mGlu_1_ ligands, and suggests that mGlu_1_ PAMs may only be effective in patients carrying *GRM1* mutations. These studies further highlight the heterogeneity of schizophrenia and the critical role of patient selection strategies in psychiatric clinical trials to match genotype with the therapy.

**Table 1:** Summary of Preclinical Efficacy of Group I mGlu Receptor Ligands

	Positive Symptom Models	Negative Symptom Models	Cognition Models
*mGlu*_*1*_ *PAMs*			
VU0483605	No effect on AHL [41]		
*mGlu*_*1*_ *NAMs*			
FTIDC	Reduced methamphetamine hyperlocomotion (MHL) [56] Ameliorated METH-induced deficits in PPI [56]		
CFMTI	Reduced MHL and NMDAR antagonist-induced hyperlocomotion (NMDAR-HL) [57] Ameliorated METH and KET-disrupted PPI [57]	Ameliorated MK-801-disrupted social interaction [57]	No effect on object location memory (OLM) [57]
*mGlu*_*5*_ *PAMs*			
CDPPB	Reduced AHL [70] Ameliorated AMP-disrupted PPI [69, 70]	Attenuated MK-801-induced decrease in sucrose preference [223]	Enhanced learning in Morris water maze (MWM) [72] Attenuated MK-801-induced deficits in cognitive flexibility [224] Improved PCP-induced deficits in novel object recognition (NOR) [225]
5PAM523	Reduced AHL [76]		
VU0409551	Reduced AHL and NMDAR-HL [81]		Enhanced contextual fear conditioning (CF) [81] Enhanced NOR [81] Enhanced working memory/executive function in the delayed non-matching to position (DNMTP) task [81] Improved contextual CF deficits in SR^−/−^ mice [81]

### mGlu_5_

In recent years, mGlu_5_ has emerged as an attractive target for the treatment of schizophrenia [59]. Similar to mGlu_1_, mGlu_5_ is primarily postsynaptic but is also located presynaptically and can be expressed on GABAergic neurons and glia (Fig. 1). In the hippocampus, prefrontal cortex (PFC), and other brain regions, mGlu_5_ plays important roles in synaptic plasticity - the strengthening or weakening of synapses in response to specific activity patterns termed long term potentiation (LTP) and long term depression (LTD), respectively [60, 61]. Early pharmacological and genetic deletion studies in mice have shown that mGlu_5_ is important in the regulation of specific domains of cognitive function [60, 61] and in behaviors relevant for the positive and negative symptoms of schizophrenia [39, 62, 63]. Interestingly, unlike mGlu_1_, early studies did not provide evidence that mGlu_5_ activation reduces dopamine release in the striatum [64, 65] thus any antipsychotic effects of mGlu_5_ activators may be independent of dopamine modulation.

Over the last two decades, a growing body of evidence suggests that selective mGlu_5_ PAMs could provide an exciting new approach for the treatment of schizophrenia [66] (Table 1). The first highly selective mGlu_5_ PAMs DFB [67] and CPPHA [68] demonstrated the viability of developing selective compounds for mGlu_5_, but lacked properties that would allow their use in vivo. The first major in vivo breakthrough came with the development of CDPPB [69], the first mGlu_5_ PAM to possess favorable DMPK properties to allow its use in rodent models [69, 70]. Subsequently, it was shown that CDPPB reverses AHL and amphetamine-induced disruption of PPI in rats, providing strong preclinical support for mGlu_5_ as a potential therapeutic for schizophrenia [70]. In more recent years, there has been tremendous success in developing a large number of structurally distinct, highly selective mGlu_5_ PAMs that have efficacy in a wide range of animal models relevant to all three symptom domains of schizophrenia [71–76].

**Fig. 2:**
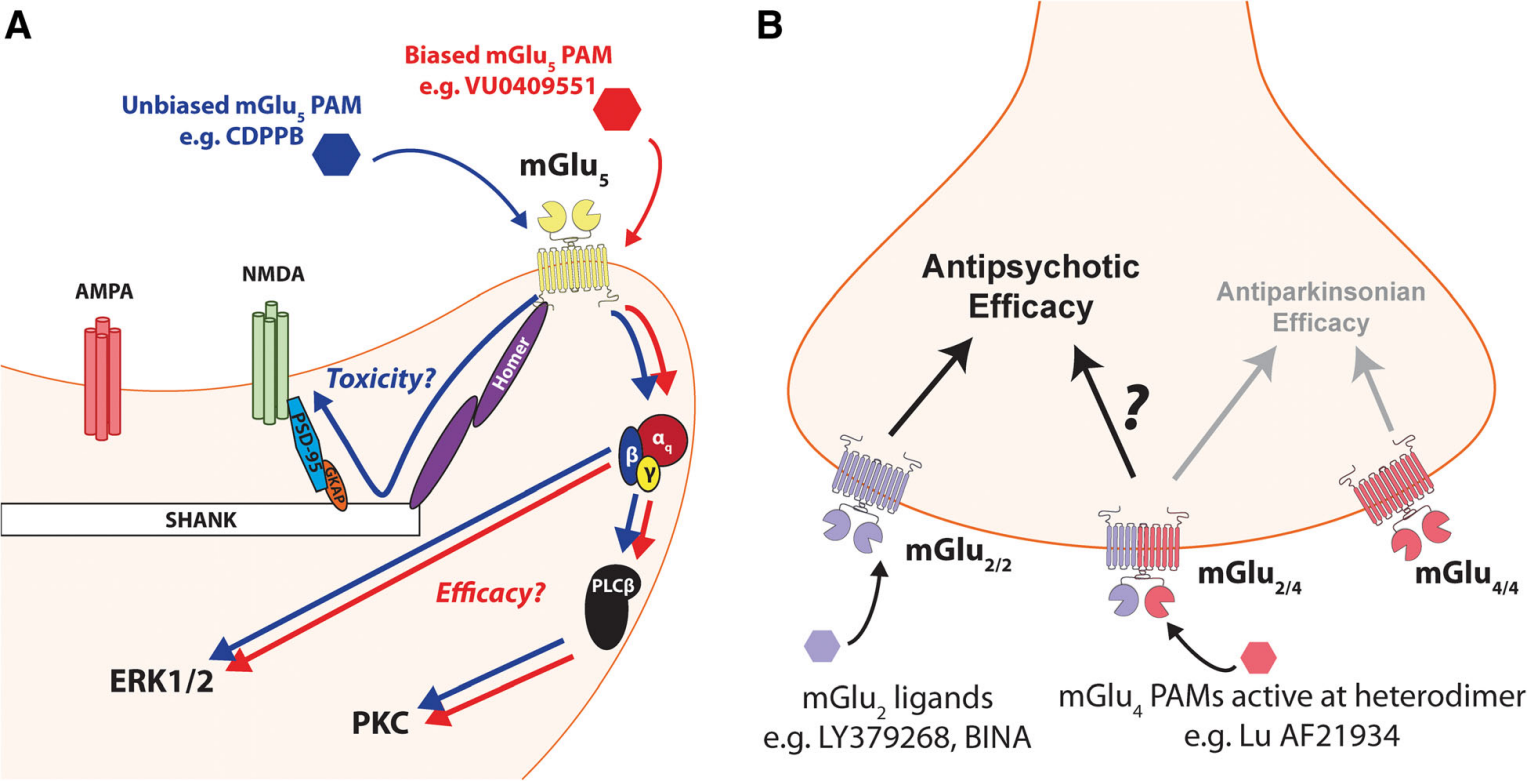
Emerging concepts in the development of mGlu receptor-targeting antipsychotic therapeutics. **a** mGlu_5_ PAMs have recently been developed that bias mGlu_5_ signaling away from NMDAR modulation but still mobilize intracellular Ca^2+^ and activate ERK1/2. The mechanism of this bias is still unclear but could involve G-protein dependent versus independent coupling of mGlu_5_ to NMDAR. The biased mGlu_5_ PAM VU0409551 retains antipsychotic-like efficacy in vivo but does not cause excitotoxicity or seizures observed with unbiased mGlu_5_ PAMs that enhance mGlu_5_-mediated modulation of NMDAR currents. This suggests that NMDAR modulation is not necessary for in vivo efficacy and that this signal bias may provide a means to overcome the NMDAR-mediated excitotoxicity that has stalled mGlu_5_ PAM development. **b** Recently, functional mGlu_2/4_ heterodimers with unique pharmacology have been identified. This suggests that actions at the mGlu_2/4_ heterodimer rather than at the mGlu_4/4_ homodimer might underlie the antipsychotic efficacy of mGlu_4_ PAMs, such as Lu AF21934, consistent with the antipsychotic-like effects of mGlu_2_-specific ligands. This remains to be tested experimentally but may provide an interesting alternative to failed mGlu_2_ clinical programs

Since mGlu_5_ can potentiate NMDAR responses in select rodent brain regions [77–79], it was initially proposed that mGlu_5_ PAMs were likely to exert their efficacy through potentiation of mGlu_5_-induced increases in NMDAR currents in forebrain regions implicated in the pathology of schizophrenia [72, 79]. Unfortunately, some mGlu_5_ PAMs, such as 5PAM523 which has efficacy in reversing AHL, appear to induce severe adverse effects including seizures and neuronal death which could be related to excessive activation of NMDAR [76, 80]. Until recently, the hypothesis that potentiation of mGlu_5_ modulation of NMDAR currents was critical for the efficacy of these compounds had not been tested. To systematically test this, a novel biased mGlu_5_ PAM, VU0409551, was developed that potentiates mGlu_5_ coupling to Gα_q_-mediated calcium mobilization and other canonical signaling pathways but does not enhance mGlu_5_ changes in NMDAR currents (Fig. 2a) [81]. Of interest, VU0409551 produces robust antipsychotic-like effects in pharmacological challenge models of positive psychotic symptoms and cognition-enhancing effects in wild-type animals [81]. VU0409551 also has robust efficacy in reversing deficits in serine racemase knockout (SR^−/−^) mice, a genetic model of NMDAR hypofunction in which the enzyme that synthesizes the NMDAR co-agonist D-serine is genetically deleted [82]. SR^−/−^ mice display deficits in synaptic plasticity and cognition [83], and recapitulate anhedonic-like symptoms, such as a blunted reward response to cocaine in an intracranial self-stimulation paradigm [84]. Interestingly, VU0409551 rescues signaling, plasticity, and cognitive deficits in this model [82], strengthening the hypothesis that biased mGlu_5_ PAMs that do not potentiate NMDAR currents still retain efficacy in rodent models relevant for schizophrenia. Furthermore, chronic administration of VU0409551 at doses over 100× those required to achieve in vivo efficacy resulted in no measureable cell death or induction of seizures [81]. In addition, separate studies revealed that eliminating allosteric agonist activity of mGlu_5_ PAMs is critical for reducing seizure liability [80]. Thus, by developing a detailed understanding of the pharmacodynamic actions of different mGlu_5_ PAMs, it may be possible to develop mGlu_5_ PAM clinical candidates that have robust efficacy but are devoid of excitotoxic adverse effects (Fig. 2a).

The mechanism by which VU0409551 exerts its antipsychotic-like and procognitive effects in animal models remains unclear. Experiments in wild-type rats suggest that the ability of VU0409551 to enhance certain forms of cognition is independent of NMDAR modulation [81]. It is possible that these effects of the PAM are due to the potentiation of mGlu_5_-mediated effects on neuronal excitability aside from NMDAR current modulation. In CA1 pyramidal cells mGlu_5_ activation suppresses the afterhyperpolarization current, thereby increasing the excitability of these neurons [79]. In these same neurons, mGlu_5_ is critical for a form of long-term plasticity at inhibitory synapses, termed inhibitory long-term depression (iLTD), and an mGlu_5_ PAM could increase hippocampal transmission via a reduction of inhibitory tone [85]. In layer V pyramidal neurons in the rodent medial PFC, mGlu_5_ activation increases neuronal excitability and spiking frequency [86, 87] as well as excitatory drive onto these neurons [88]. One hypothesis is that VU0409551 exerts its procognitive effects, especially the augmentation of PFC-dependent recognition memory, working memory, and executive function [81], via increased PFC pyramidal neuron activity but this remains untested.

In vitro assays indicate that VU0409551 exerts both PAM and robust agonist activity with respect to mGlu_5_-mediated extracellular-signal regulated kinase (ERK) activity. This is in agreement with the ability of VU0409551 to enhance LTD at the Schaffer Collateral-CA1 (SC-CA1) synapse induced by the group I mGlu receptor agonist DHPG, a form of plasticity that involves rapid protein synthesis and ERK activation [89–91]. Additionally, augmentation of early-phase LTP (E-LTP; < 3 h) by mGlu_5_ PAMs may require NMDAR current potentiation, explaining why VU0409551 does not augment E-LTP. While E-LTP is not dependent on ERK activity nor protein synthesis, late-phase LTP (>3 h) is ERK-dependent [92], can be enhanced by mGlu_5_ PAMs [93] and is closely linked to long term memory consolidation. Therefore, VU0409551 via its positive effects on ERK activation may exert its procognitive effects by potentiating late-phase LTP, although this remains to be tested experimentally.

Interestingly, VU0409551 is able to rescue deficits in hippocampal E-LTP in SR^−/−^ mice without any augmentation in littermate controls [82]. This effect also correlates with the ability of VU0409551 to enhance NMDAR synaptic responses exclusively in the knockout mice. How VU0409551 exerts these effects in animals with marked NMDAR hypofunction [94] but not in wildtype animals remains to be determined. It is still unclear how prototypical mGlu_5_ PAMs enhance NMDAR function in wild-type animals [68, 81] as studies have implicated both G-protein-dependent [95–98] and G-protein-independent [99–102] pathways in the mGlu_5_-NMDAR interaction (Fig. 2a). Therefore, the actions of VU0409551 in SR^−/−^ mice could involve a rearrangement of the postsynaptic density to prefer G-protein-independent mGlu_5_-mediated NMDAR current enhancement or differential spatial and/or temporal coupling of mGlu_5_ to G-protein-dependent downstream effectors that could augment NMDARs such as PKC and CaMKII. Future work is still needed to determine how mGlu_5_ PAMs enhance NMDAR function in wild-type animals and schizophrenia-like animal models.

## Group II mGlu receptors (mGlu_2_ & mGlu_3_)

The group II mGlu receptors, mGlu_2_ and mGlu_3_ [103], are expressed presynaptically [22] outside of the active zone on pre-terminal regions of axons where they can be activated by astrocytic glutamate release or excessive synaptic glutamate [104] (Fig. 1). mGlu_3_ is also found postsynaptically as well as on astrocytes where it mediates neuroprotective effects [104] and participates in astrocytic-neuronal communication [105, 106] (Fig. 1). Group II mGlu receptors traditionally couple to the Gα_i/o_ subunits of G-proteins, leading to inhibition of adenylyl cyclase and other signaling pathways [22].

Due primarily to their inhibition of neurotransmitter release from glutamatergic, GABAergic, and neuromodulatory (dopaminergic, noradrenergic, etc.) presynaptic terminals (Fig. 1), the group II mGlu receptors have attracted considerable attention as potential targets for novel antipsychotics. mGlu_2/3_ activation has been shown to reduce extracellular dopamine efflux in the nucleus accumbens [48, 64], the substantia nigra [47], and recently afferent-driven dopamine release in the dorsal striatum [107]. Additionally, elevated post-mortem glutamatergic markers are observed in the cortex of schizophrenia patients [108], commonly attributed to NMDAR dysfunction on GABAergic interneurons leading to an overall net disinhibition [10, 109, 110]. Furthermore, in both humans and animal models, NMDAR antagonist psychotomimetics increase glutamatergic transmission in the prefrontal cortex and increase CNS glutamate levels [10, 111–114]. mGlu_2/3_ activation can inhibit glutamate release, therefore providing rationale for the development of agonists or potentiators of mGlu_2/3_ to reduce the excessive glutamatergic tone in the brain of patients with schizophrenia [115].

Directly relevant to NMDAR hypofunction, postsynaptic mGlu_2/3_ activation can also enhance NMDAR currents in CA1 hippocampal pyramidal cells via Src kinase [116] as well as in PFC pyramidal cells via PKC [117] and SNARE-dependent mechanisms [118]. It is currently unclear whether this enhancement of NMDAR currents contributes to the antipsychotic efficacy of mGlu_2/3_ activators but it is interesting that compounds targeting these receptors could provide a two-pronged approach: normalizing both NMDAR function and excessive glutamate levels in schizophrenia.

Of further importance, a functional heteromeric complex between the 5-HT_2A_ serotonin receptor (5-HT_2A_R) and mGlu_2_ has been postulated based on biochemical, behavioral, and pharmacological data [119–121]. 5-HT_2A_R agonists such as psychedelic hallucinogens typically induce a psychotomimetic state in humans [122] similar to observations with NMDAR antagonists. Activation of 5-HT_2A_R enhances thalamocortical neurotransmission in rodents [123, 124] and this effect is antagonized by activation of group II mGlu receptors [125]. Orthosteric agonists of mGlu_2/3_ functionally antagonize 5-HT_2A_ receptor signaling [119], therefore activators of mGlu_2_ may possess antipsychotic properties similar to atypical antipsychotics that partially rely on 5-HT_2A_R antagonism for their efficacy. An issue this raises for clinical trials is that antagonism of 5-HT_2A_ with atypical antipsychotics has been shown to downregulate expression of *GRM2* (the gene encoding mGlu_2_) in rodents and decrease positive epigenetic markers of *GRM2* expression in both mice and humans [126]. Based on these data, it may be essential to stratify future patient populations based on prior use of atypical serotonergic antipsychotics to ensure adequate target engagement and therapeutic efficacy of mGlu_2_ ligands.

### mGlu_2/3_ agonists

Interest in targeting mGlu_2/3_ for the treatment of schizophrenia began with the initial findings that the selective group II mGlu receptor orthosteric agonists LY354740 and LY379268 can reverse the ability of NMDAR antagonists to induce hyperlocomotion, stereotypies, deficits in working memory, cortical glutamate efflux, and increased firing of PFC neurons in rats [114, 127, 128] (Table 2). One of the most intriguing results of these studies was that activation of group II mGlu receptors had no effect on the NMDAR antagonist-induced rise in extracellular dopamine in the CNS [127]. Despite this, group II mGlu receptor agonists still retained antipsychotic-like efficacy in a range of animal models, suggesting the possibility of treating psychosis without the extrapyramidal or other adverse effects associated with dopaminergic antipsychotics. On the other hand, when the mGlu_2/3_ agonist LY404039 was administered to rats, it caused an increase in extracellular dopamine, the dopamine metabolites 3,4-dihydroxyphenylacetic acid (DOPAC) and homovanillic (HVA), and the serotonin metabolite 5-hydroxyindoleacetic acid (5-HIAA) in the PFC [129, 130]. In contrast to the hyperdopaminergic state in the striatum thought to underlie the positive symptoms of schizophrenia, it is hypothesized that the negative symptoms of the disease are contributed to by a dearth of dopamine and serotonin release in cortical regions [131–134]. Therefore, the increased dopamine as well as dopamine and serotonin turnover observed in the rat PFC in response to LY404039 [129] may predict efficacy in treating some aspects of the negative symptoms of schizophrenia.

**Table 2:** Summary of Preclinical Efficacy of Group II mGlu Receptor Ligands

	Positive Symptom Models	Negative Symptom Models	Cognition Models
*mGlu*_*2/3*_ *agonists*			
LY354740	Reduced NMDAR-HL [127, 226] No effect on AHL [226, 227] Reduced NMDAR antagonist-induced stereotypies [127, 128] No effect on apomorphine-disrupted PPI [227] Inhibited DOI-induced head twitches [228]	Reduced PCP-induced deficits in social interaction [229]	Reduced PCP-induced deficits in the discrete-trial delayed alternation (DTDA) task [127] Impaired performance in delayed matching to position (DMTP) and DNMTP tasks [143]
LY379268	Reduced NMDAR-HL and AHL [141, 226, 230] No effect on PCP- or AMP-disrupted PPI [141] Inhibited DOI-induced head twitches [228] Reduced prenatal restraint-induced HL/PPI deficits [231] Reversed postnatal isolation-induced HL/PPI deficits [232]	Reduced MK-801-increase in immobility in forced swim test (FST) [230] Attenuated MK-801-induced deficits in social interaction [233, 234] Rescued prenatal restraint-induced deficits in social interaction [231]	Reduced PCP-induced increase in errors, but not accuracy, in the 5-choice serial reaction time task (5CSRT) [235] Exacerbated PCP-induced deficits in 5CRST task [236] Rescued MK-801-induced deficits in NOR [234] Reversed postnatal isolation-induced deficits in NOR [232]
*mGlu*_*2*_ *PAMs*			
LY487379	Reduced NMDAR-HL and AHL [141] Attenuated AMP but not PCP-disrupted PPI [141]	Reduced PCP-induced deficits in social interaction [229]	Promoted cognitive flexibility in attentional set-shift task (ASST) [237]
BINA	Reduced NMDAR-HL [149, 230] No effect on AHL [149] Reduced PCP-disrupted PPI [149] Reduced DOB-induced head twitches [150]	Reduced MK-801-induced increased immobility in the FST [230]	
TASP0443294	Reduced MHL [156]	Rescued MK-801 induced social memory deficits [156]	
JNJ-40411813/ADX71149	Reduced NMDAR-HL [158] No effect on AHL [158] Inhibited DOM-induced head twitches [158]		
SAR218645	No effect on NMDAR-HL or AHL [160] No effect on hyperactivity in DAT^−/−^ and NR1^neo−/−^ mice [160] Reduced DOI-induced head twitches [160]		Reversed MK-801-induced deficits in NOR [160] Attenuated working memory deficits in Y-maze test in NR1^neo−/−^ mice [160]

Based on the extensive preclinical evidence in support of mGlu_2/3_ agonists as novel antipsychotics, Eli Lilly & Co. progressed LY2140023 monohydrate (pomaglumetad methionil; prodrug of the active mGlu_2/3_ agonist LY404039) into clinical trials and demonstrated safety and tolerability in humans [135]. In a 4-week multicenter proof-of-concept phase II clinical trial of 196 patients randomly assigned to receive LY2140023, olanzapine, or placebo, LY2140023 showed statistically significant improvements in positive and negative symptoms (assessed by the Positive And Negative Symptom Scale, PANSS) relative to placebo and was comparable to the currently approved atypical antipsychotic olanzapine [136]. Most excitingly, this study found that LY2140023 was well-tolerated and did not produce any EPS or elevated prolactin levels [136].

Following these promising initial results, a second 4-week phase II dose-ranging study found that neither LY2140023 nor olanzapine was more efficacious than placebo. Thus, the results were inconclusive due to an abnormally high placebo effect [137]. In a subsequent 24 week phase II study LY2140023 was found to significantly reduce PANSS scores over the 24-week period but from weeks 16 to 24 it was less effective than the current standard of care group (treatment with olanzapine, aripiprazole, or risperidone) [138]. Discouragingly, in a larger phase II trial of 1013 patients, LY2140023 failed to show improvements in PANSS total score compared to placebo, while the atypical antipsychotic risperidone significantly separated from placebo [139]. A separate phase 1b study found that LY2140023 also failed to demonstrate efficacy in alleviating negative symptoms when administered concurrently with atypical antipsychotics although this has yet to be analyzed post-hoc based on prior patient antipsychotic use [140]. In response to these undesirable larger-scale clinical trial results, Eli Lilly and Co. terminated the development of LY2140023.

### mGlu_2_ PAMs

Although preclinical studies with group II mGlu agonists appeared promising, chronic administration of group II mGlu receptor agonists resulted in robust tolerance and loss of their ability to reverse amphetamine- and PCP-induced hyperlocomotion [141]. It is possible that this contributed to the lack of reliable clinical efficacy outlined above. Additionally, group II mGlu receptor agonists can impair working and spatial memory in rodent models [142, 143]. However, studies with mGlu_2_ and mGlu_3_ knockout mice suggest that the reversal of amphetamine- and PCP-induced hyperlocomotion by group II mGlu agonists was dependent on activation of mGlu_2_, not mGlu_3_ [144, 145], prompting the development of mGlu_2_ selective PAMs. By potentiating responses to endogenous glutamate, it is possible that mGlu_2_ PAMs could reverse excessive glutamatergic signaling only at synapses where this pathophysiology is present, potentially avoiding the tolerance and cognition-impairing effects seen with orthosteric agonists and providing an alternative path forward for therapeutics targeting these receptors.

Two prototypical mGlu_2_ PAMs, LY487379 [146, 147] and biphenyl-indanone A (BINA) [148, 149], showed efficacy in reversing amphetamine- and PCP-induced hyperlocomotion and disruptions in PPI (Table 2). Furthermore, BINA was able to attenuate the serotonin-induced increase in excitatory transmission in the PFC and reduce head twitch behavior induced by the 5-HT_2A_ receptor agonist (−)-DOB [150]. Therefore, mGlu_2_ PAMs were efficacious in dopaminergic, glutamatergic, and serotonergic pharmacological models of the positive symptoms of schizophrenia. These studies provided foundational research which motivated multiple drug discovery programs to develop selective mGlu_2_ PAMs [151–155] that have efficacy in animal models of schizophrenia including TASP0443294 [156], JNJ-40411813/ADX71149 [157, 158], AZD8529 [159], and SAR218645 [160] (Table 2).

TASP0443294 dose-dependently attenuated methamphetamine-induced hyperlocomotion, MK-801-induced deficits in social memory, and ketamine-induced increases in cortical gamma power, as well as reducing the duration of REM sleep in rats [156]. JNJ-40411813/ADX71149 also dose-dependently inhibited PCP- and scopolamine-induced but intriguingly not amphetamine-induced hyperlocomotion. Furthermore, JNJ40411813/ADX71149 reduced brain glucose metabolism induced by the NMDAR antagonist memantine and head twitch response induced by the 5-HT_2A_ agonist DOM [158]. Recently, SAR218645 was shown to reduce DOI-induced cortical glutamate release and head twitch behavior but had no effect in pharmacological or genetic dopaminergic and glutamatergic models of the positive symptoms of schizophrenia [160]. SAR218645 did improve MK-801-induced short-term episodic memory as well as working memory deficits in GluN1 knockdown mice, providing the first evidence of cognition-enhancing effects of mGlu_2_ PAMs in a genetic model of schizophrenia [160]. Based on these results, the authors suggested that mGlu_2_ PAMs with profiles like SAR218645 might be efficacious in treating the cognitive deficits in schizophrenia but not the positive symptoms [160].

To date, two mGlu_2_ PAMs have progressed to clinical trials: JNJ40411813/ADX71149 [161] and AZD8529 [159]. Phase I assessment in healthy volunteers indicated that JNJ40411813 was generally well tolerated in healthy men and women--with adverse events such as ataxia and somnolence emerging only at high doses [161]. However, secondary measures of cognition endpoints suggested that the mGlu_2_ PAM decreased accuracy in an attention task in healthy men. Although, JNJ40411813 did trend to reduce cognitive deficits in attention and episodic memory precipitated by smoking withdrawal in a subpopulation of healthy volunteers, this was not statistically significant compared to placebo. Promisingly in a proportion of volunteers, 500 mg JNJ40411813 reduced the increase in Brief Psychiatric Rating Scale (BPRS) total score and negative symptom score induced by a low dose of (S)-ketamine [161]. Based on its tolerability and promising initial results in the ketamine challenge, it will be interesting to see if Johnson & Johnson will progress the compound further.

Recently, the phase II trial results of AstraZeneca’s mGlu_2_ PAM AZD8529 were disclosed [159]. Despite being well tolerated with mild adverse events, AZD8529 did not show any improvements in PANSS total score or PANSS positive and negative subscale scores compared to placebo. While AZD8529 did not produce any extrapyramidal side effects or elevation of prolactin (an effect observed with the comparator risperidone) it failed to demonstrate efficacy in this study of 104 patients with schizophrenia [159]. Possible explanations for this lack of efficacy include lack of sufficient target engagement and the use of a less symptomatic patient population. However, CNS activity suggesting target engagement was subsequently validated using fMRI, and risperidone significantly improved PANSS scores compared to placebo, suggesting that this mGlu_2_ PAM may lack sufficient efficacy even at doses that provide CNS effects [159].

Together with the disappointing results of the group II agonist LY2140023 trials, there is a significant discrepancy between these preclinical data implicating glutamatergic dysfunction and mGlu_2_ agonist or PAM efficacy and these clinical data. This could be in part due to improper patient selection, as hyperactivity of cortical regions correlates with psychosis only early on in disease progression [162, 163]. Furthermore, since atypical antipsychotics may decrease mGlu_2_ levels via the 5HT_2A_/mGlu_2_ heteromer [126], lower receptor levels might contribute to the lack of efficacy in the patient populations used in either study. While an intriguing possibility, this remains to be tested.

### mGlu_3_

While pharmacological manipulation of group II mGlu receptors was based on the normalization of aberrant glutamatergic signaling downstream of NMDAR hypofunction, single nucleotide polymorphisms (SNPs) in the *GRM3* gene encoding mGlu_3_ have been associated with schizophrenia in multiple studies [164–167]. No studies to date have found statistically significant associations with *GRM2* SNPs [168, 169]. The association between *GRM3* and schizophrenia has been extensively reviewed in the past, with certain SNPs associated with deficits in working and episodic memory [166]. More recently, a large-scale genome-wide association study of almost 37,000 patients with schizophrenia identified the *GRM3* locus, as well as 108 other loci, associated with schizophrenia [170], supporting the idea that mGlu_3_ may be a viable target along with mGlu_2_, despite the antipsychotic-like efficacy of mGlu_2_ specific potentiators in rodent models.

Supporting this, a recent study using the mGlu_2_ agonist/mGlu_3_ antagonist LY395756 [171] showed that mGlu_2_ agonism was sufficient to enhance NMDAR function but the combination of mGlu_2_ agonism and mGlu_3_ antagonism could not reverse MK801-induced deficits in working memory [172]. This is consistent with the finding that mGlu_3_ is required for a form of LTD in the mouse PFC and that a selective mGlu_3_ negative allosteric modulator impairs PFC-dependent cognition [173]. Based on these findings and the neuroprotective role of mGlu_3_ [174–177], agonism or enhancement of mGlu_3_ signaling may provide pro-cognitive benefits in addition to ameliorating some of the neuroinflammatory pathology seen in schizophrenia [178, 179]. Finally, it has recently been reported that mGlu_3_ activation can positively modulate mGlu_5_ signaling [180], providing a potential mechanism to enhance NMDAR function (via mGlu_3_-mGlu_5_-NMDAR interactions) and consequently provide both antipsychotic and pro-cognitive efficacy. Though this hypothesis remains to be tested, the biological role and preclinical pharmacology indicate that enhancement of mGlu_3_ might be a promising strategy for the treatment of schizophrenia, especially with potential for improving cognitive disturbances in patients with schizophrenia.

## Group III mGlu receptors (mGlu_4_, mGlu_7_, & mGlu_8_)

The group III mGlu receptors are grouped based on high sequence homology and consist of mGlu_4_ [103], mGlu_6_ [181], mGlu_7_ [182], and mGlu_8_ [22, 183]. mGlu_6_ is expressed exclusively in the retina, whereas the other group III mGlu receptors are primarily expressed in the CNS [181]. Similar to group II, group III mGlu receptors canonically signal via the Gα_i/o_ subunits of the heterotrimeric G-protein complex, leading to inhibition of adenylyl cyclase and cAMP production [22]. Activation of group III mGlu receptors can also regulate neurotransmitter release via activation/inhibition of different ion channels and G_βY_-dependent inhibition of vesicular fusion [184]. Akin to the group II mGlu receptors, the therapeutic promise of group III mGlu receptor activators or potentiators arises from their hypothesized ability to ameliorate the hyperglutamatergic state proposed to take place in schizophrenia. Also, group III mGlu receptor activation reduces dopamine release in the nucleus accumbens [64] but more work is needed to ascertain if this reduction of dopamine release would contribute to potential antipsychotic efficacy of group III mGlu receptor agonists and/or PAMs.

### mGlu_4_

mGlu_4_ is expressed predominantly on presynaptic glutamatergic and GABAergic terminals [22] (Fig. 1). In multiple immunohistochemistry studies, mGlu_4_ has been shown to localize to the presynaptic active zone, where it is situated to function as an auto- and heteroreceptor upon the release of glutamate into the synaptic cleft [185, 186]. mGlu_4_ is highly expressed in the cerebellum, moderately expressed in the olfactory bulb and thalamus, and lowly expressed in the hippocampus and the striatum [187]. Likely due to high levels of mGlu_4_ in the cerebellum, mGlu_4_ KO mice have deficits in cerebellar synaptic plasticity and impaired ability to learn complicated motor tasks [188]. Mice lacking mGlu_4_ also display deficits in spatial reversal and long-term memory [189], indicating a role of mGlu_4_ in cognition and cognitive flexibility, both of which are impaired in schizophrenia.

Multiple studies suggest that activation of mGlu_4_ may have antipsychotic-like effects in rodent models (Table 3). The pan-group III agonist ACPT-I reduced PCP- and amphetamine-induced hyperlocomotion as well as DOI-induced head twitches [190], and these actions of ACPT-I are also observed with mGlu_4_-selective agonists, LSP1-2111 [191] (>30-fold selective for mGlu_4_ vs. mGlu_8_) and LSP4-2022 [192] (>100-fold selective for mGlu_4_ vs. mGlu_7_; >300-fold vs. mGlu_8_). In addition to efficacy in models of the positive symptoms of schizophrenia, both LSP1-2111 and LSP4-2022 have efficacy in models of negative symptoms and cognitive deficits [191, 192]. Furthermore, the mGlu_4_-selective PAMs Lu AF21934 [193], Lu AF32615 [194], and ADX88178 [195] displayed similar promise in models of all three symptom clusters of schizophrenia [196, 197], providing further support for potential therapeutic utility of selective mGlu_4_ activators.

**Table 3:** Summary of Preclinical Efficacy of Group III mGlu Receptor Ligands

	Positive Symptom Models	Negative Symptom Models	Cognition Models
*Group III agonist*			
ACPT-I	Reduced NMDAR-HL and AHL [190] Reduced DOI-induced head twitches [190]		
*mGlu*_*4*_ *agonists*			
LSP1-2111	Reduced NMDAR-HL and AHL [191] Reduced DOI-induced head twitches [191]		
LSP4-2022	Reduced NMDAR-HL [192] Reduced DOI-induced head twitches [192]	Reduced MK-801-induced deficits in social interaction [192]	Reduced MK-801-induced deficits in NOR [192]
*mGlu*_*4*_ *PAMs*			
Lu AF21934	Reduced NMDAR-HL and AHL [196, 238] Reduced DOI-induced head twitches [196, 238]	Reduced MK-801-induced deficits in social interaction [196, 238]	Rescued MK-801-induced deficits in the delayed spatial alternation task [196] Reduced MK-801-induced deficits in NOR [238]
Lu AF32615	Reduced NMDAR-HL and AHL [196] Reduced DOI-induced head twitches [196]	Reduced MK-801-induced deficits in social interaction [196]	Rescued MK-801-induced deficits in the delayed spatial alternation task [196]
ADX88178	Reduced NMDAR-HL [197] Reduced DOI-induced head twitches [197]	Reduced immobility in FST [197]	
*mGlu*_*7*_ *agonist*			
AMN082	Exacerbated NMDAR-HL [191] No effect on AHL [191] Exacerbated DOI-induced head twitches [191]		
*mGlu*_*8*_ *agonist*			
(S)-3,4-DCPG	No effect on NMDAR-HL or AHL [221]		

Interestingly, recent studies raise the possibility that some of the in vivo actions of mGlu_4_ agonists or PAMs could be mediated by actions on mGlu_2/4_ heterodimers (Fig. 2b). While mGlu receptors are thought to function primarily as homodimers [198], in recent years it has become apparent that functional mGlu heterodimers exist and can have unique profiles in terms of altered signaling and pharmacology [199–201]. Recent studies reveal that a heterodimer between mGlu_2_ and mGlu_4_ exists and displays unique pharmacology compared to mGlu_2_ or mGlu_4_ homodimers [201, 202]. Interestingly, mGlu_2/4_ heterodimers are activated by orthosteric agonists of either mGlu_2/3_ or mGlu_4_ [201]. Furthermore, Lu AF21934, an mGlu_4_ PAM that has efficacy in rodent models of antipsychotic-like effects, has robust efficacy as an mGlu_2/4_ heterodimer PAM (Fig. 2b). Thus, while studies have yet to directly test the hypothesis that mGlu_2/4_ heterodimers are involved in the antipsychotic-like effects of these compounds, it will be important to consider this possibility in future studies.

### mGlu_7_

A polymorphism in the *GRM7* gene encoding mGlu_7_ that reduced transcription in vitro was found to be positively associated with schizophrenia in a large Japanese cohort [203], indicating that hypofunction of mGlu_7_ may contribute to this disorder. However, at present, few studies have focused on a potential role of mGlu_7_ in the pathophysiology of schizophrenia. Interestingly, mGlu_7_ exhibits the widest expression of group III receptors [187, 204], with high expression in cortex, hippocampus, and other forebrain regions [205]. Studies with mGlu_7_ KO mice demonstrated a role of mGlu_7_ in hippocampal short-term plasticity [206], amygdala-dependent learning processes [206], short-term working memory [207, 208], and extinction learning [208, 209]. Also, activation of mGlu_7_ reduces glutamatergic neurotransmission at the SC-CA1 synapse in the hippocampus [210, 211] and acts as a heteroreceptor (Fig. 1) to modulate GABA release and the induction of LTP at SC-CA1 [212]. Thus, selective activators of mGlu_7_ have the potential to enhance some aspects of hippocampal-dependent cognitive function. In addition, evidence suggests that mGlu_7_ activation reduces thalamocortical neurotransmission [213], a circuit thought to be overactive in schizophrenia [214]. However, the mGlu_7_ allosteric agonist AMN082 [215] exacerbates MK-801-induced hyperlocomotion and DOI-induced head twitches [191] (Table 3). While this may be due to off-target effects of AMN082 or its metabolites in vivo [216], these pro-psychotic effects were absent in mGlu_7_ KO mice [191] suggesting that they are mediated by mGlu_7_. It remains to be seen if the same pro-psychotic effects are observed using selective PAMs and future studies are needed to fully evaluate the potential utility of mGlu_7_ agonists or PAMs in schizophrenia-related models.

### mGlu_8_

mGlu_8_ is widely expressed throughout the brain, although at relatively low levels compared to other group III mGlu receptors [22]. Like mGlu_4_ and mGlu_7_, mGlu_8_ is expressed in the presynaptic active zone of mainly glutamatergic synapses [185, 217] (Fig. 1) where it functions to modulate neurotransmitter release. It has also been identified in the postsynaptic compartment in the retina, medulla, and periphery [205]. mGlu_8_ has been shown to function as an autoreceptor at the lateral perforant path synapse in the dentate gyrus [218], thus gating glutamatergic transmission into the hippocampus. Consistent with this, mGlu_8_ KO mice display deficits in hippocampal-dependent learning [219]. Additionally, mGlu_8_ suppresses glutamatergic input into the bed nucleus of the stria terminalis (BNST) implicating a role for this receptor in anxiety and stress [220], consistent with results observed in the mGlu_8_ KO mice [221]. Similar to both mGlu_4_ and mGlu_7_, the neuromodulatory role of mGlu_8_ in brain regions implicated in learning and memory suggests that mGlu_8_ ligands could be beneficial in treating the cognitive deficits in patients with schizophrenia.

In studies investigating the potential antipsychotic efficacy of targeting mGlu_8_, researchers from GlaxoSmithKline found that the relatively selective orthosteric mGlu_8_ agonist (S)-3,4-DCPG [222] was unable to reverse PCP-induced or amphetamine-induced hyperactivity in Sprague-Dawley rats [221] (Table 3). Furthermore, mGlu_8_ KO mice had no significant deficits in PPI and thus it was concluded that mGlu_8_ does not appear to be involved in the etiology of schizophrenia nor does it appear to be a potential target for a novel antipsychotic [221]. This may be true with respect to positive symptoms but, based on the role mGlu_8_ plays in hippocampal neurotransmission [218, 221] it is still possible that agonists or potentiators of mGlu_8_ can have cognitive-enhancing properties. While exciting, this remains to be tested.

## Conclusion

Extensive preclinical evidence has implicated all three groups of mGlu receptors as viable targets for the development of novel therapeutics for the treatment of schizophrenia. Agonists and subtype-selective PAMs for these receptors have efficacy in dopaminergic, serotonergic, and glutamatergic models of the positive and negative symptoms of the disease, and also demonstrate nootropic, or cognition-enhancing effects (Tables 1, 2, and 3). Based on preclinical findings, mGlu receptor modulators have the potential to be major improvements over currently approved dopaminergic and serotonergic antipsychotics. Negative results in clinical trials for the group II agonist pomaglumetad (LY2140023) and the mGlu_2_ PAM AZD8529 are disappointing but could be explained by patient selection issues. Potential future trials with patients selected based on stage of the disease and prior antipsychotic usage may yield different results in light of our advanced knowledge of the pathophysiology of schizophrenia. These negative clinical results suggest that there are still important gaps in our knowledge of how to translate preclinical results into clinical efficacy in schizophrenia. Taken together, these studies point to potential challenges in selecting the most appropriate patient populations for evaluating different mechanisms for improving different symptoms observed in schizophrenia patients.

In addition to potential antipsychotic efficacy, selective mGlu receptor ligands could provide cognition-enhancing effects targeting a major unmet need of this disorder. To this end, the key role of mGlu_5_ in hippocampal and cortical plasticity suggests that mGlu_5_ PAMs might have their greatest effects on cognition. Furthermore, the interaction between mGlu_3_ and mGlu_5_ and the involvement of mGlu_3_ in cortical plasticity suggests that potentiators of mGlu_3_ may also exert cognition-enhancing effects. Lastly, the notion that mGlu receptor ligands would provide a powerful cognition-enhancing approach to mitigate the deficits observed in schizophrenia is further evidenced by the mGlu_2_ PAM SAR218645 improving learning and memory in rodent models of schizophrenia as well as the preclinical cognition-enhancement observed with mGlu_4_ agonists and PAMs.

Of particular importance to future development efforts, both the discovery of biased PAMs (Fig. 2a) and the existence of mGlu heterodimers with distinct pharmacology (Fig. 2b) could provide novel approaches to optimize efficacy while avoiding toxic or other adverse effects. Although relatively unexplored, these emerging concepts in the pharmacology and biology of mGlu receptors provide a new path forward in the face of negative clinical results.

In conclusion, the metabotropic glutamate receptors represent a large group of promising targets for novel therapeutics to treat all three symptom domains of schizophrenia. While many discovery efforts are still in preclinical phases of development, they have yielded several subtype-selective tool compounds with minimal adverse effect profiles and promising preclinical efficacy. These compounds provide the unprecedented opportunity to further our fundamental understanding of the therapeutic role of mGlu receptor modulation in schizophrenia and represent a potential breakthrough in treating patients suffering from this disorder.

## Abbreviations

5CSRT: 5-choice serial reaction time
5-HIAA: 5-Hydroxyindoleacetic acid
5-HT_2A_R: Serotonin (5-hydroxytryptamine) 2A receptor
AHL: Amphetamine-induced hyperlocomotion
AMP: Amphetamine
AMPA: α-amino-3-hydroxy-5-methyl-4-isoxazolepropionic acid
ASST: Attentional set-shift task
BNST: Bed nucleus of the stria terminalis
BPRS: Brief Psychiatric Rating Scale
CF: Conditioned fear
CNS: Central nervous system
DAG: Diacylglycerol
DMPK: Drug metabolism and pharmacokinetics
DMTP: Delayed matching to position
DNMTP: Delayed non-matching to position
DOPAC: 3, 4-Dihydroxyphenylacetic acid
DTDA: Discrete-trial delayed alternation
EPS: Extrapyramidal side effects
FST: Forced swim test
GABA: γ-amino butyric acid
GPCR: G-protein coupled receptor
HL: Hyperlocomotion
HVA: Homovanillic Acid
IP_3_: inositol triphosphate
KET: Ketamine
KO: Knockout
LTD: Long term depression
LTP: Long term potentiation
METH: Methamphetamine
mGlu: Metabotropic glutamate
MHL: Methamphetamine-induced hyperlocomotion
MWM: Morris water maze
NAM: Negative allosteric modulator
NMDAR: N-methyl-d-aspartate receptor
NMDAR-HL: NMDAR antagonist-induced hyperlocomotion
NOR: Novel object recognition
OLM: Object location memory
PAM: Positive allosteric modulator
PANSS: Positive and Negative Symptom Scale
PCP: Phencyclidine
PFC: Prefrontal cortex
PKC: Protein kinase C
PPI: Prepulse inhibition
SNP: Single nucleotide polymorphism
SR^−/−^: Serine racemase knockout
